# FUNCTIONAL CAPACITY IN PATIENTS WITH KNEE OSTEOARTHRITIS: CROSS-SECTIONAL STUDY

**DOI:** 10.1590/1413-785220243201e272993

**Published:** 2024-05-06

**Authors:** Edinilson Bertoldo da Silva, Camila Vitelli Molinari, Claudio Cazarini, Vera Lúcia dos Santos Alves

**Affiliations:** 1. Irmandade da Santa Casa de Misericordia de Sao Paulo, Hospital Sao Luiz Gonzaga, Serviço de Fisioterapia, Sao Paulo, SP, Brazil.; 2. Irmandade da Santa Casa de Misericordia de Sao Paulo, Hospital Central, Serviço de Fisioterapia, Sao Paulo, SP, Brazil.; 3. Irmandade da Santa Casa de Misericordia de Sao Paulo, Faculdade de Ciencias Medicas da Santa Casa de São Paulo, Sao Paulo, SP, Brazil.

**Keywords:** Osteoarthritis, Knee, Walk Test, Functional Status, Physical Functional Performance, Comorbidity, Osteoartrite do Joelho, Teste de Caminhada, Estado Funcional, Desempenho Físico Funcional, Comorbidade

## Abstract

Knee osteoarthritis (KOA) is a disabling inflammatory disease that makes walking and activities of daily living difficult. This condition can reduce functional capacity and increase the risk for surgery. Objective: To know the functional capacity of patients with KOA evaluated by the six-minute walk test (6MWT). Method: This cross-sectional study evaluated age, gender, weight, BMI, pain (VAS 90-100), physical disability (WOMAC 0-96), degree of joint damage by radiographic imaging, and 6MWT. Results: A total of 176 patients referred by Orthopedics were evaluated, with the inclusion of 164 participants. The mean age was 61.89 ± 10.62 years, 81% women, 67% with cardiovascular disease, hypertension and/or diabetes, 81% obese, with moderate pain (VAS 47.74 ± 29.27) and according to WOMAC, most had severe or very severe disability. The distance covered in the 6MWT was 354.03 ± 102.03m, 67% of the predicted distance. The maximum heart rate achieved was 107.27 ± 17.71 bpm, which characterizes 68% of the predicted by age. Only 12% of the sample showed a marked drop in oxygenation in the 6MWT and 40% had a recovery heart rate in the 1st minute below 15 bpm. Conclusion: Patients with KOA, who were evaluated by the 6MWT, have low functional capacity and physical deconditioning. ***Level of Clinical Evidence III, Case Control Study.***

## RESUMO

*A osteoartrite de joelho (OAJ) é uma doença inflamatória incapacitante que dificulta as atividades de vida diária do indivíduo. Esta condição pode reduzir a capacidade funcional e aumentar o risco de cirurgia, caso necessária. Objetivo: Conhecer a capacidade funcional de pacientes com OAJ, avaliada pelo teste de caminhada de seis minutos (TC6M). Métodos: Neste estudo transversal, avaliamos idade, sexo, peso, índice de massa corporal (IMC), dor (escala visual analógica – EVA 90-100), incapacidade física (Western Ontario and McMaster Universities Osteoarthritis Index – WOMAC 0-96), grau de lesão articular por imagem radiográfica e TC6M. Resultados: Foram avaliados 176 pacientes encaminhados pela Ortopedia, com a inclusão de 164 participantes. Na amostra, verificou-se: média de idade de 61,89 ± 10,62 anos; 81% de mulheres; 67% com doença cardiovascular, hipertensão e/ou diabetes; 81% de obesos; com dor moderada (EVA 47,74 ± 29,27); e, segundo WOMAC, maioria com incapacidade intensa ou muito intensa. A distância percorrida no TC6M foi 354,03 ± 102,03 m, sendo esse valor 67% da distância prevista. A frequência cardíaca máxima alcançada foi de 107,27 ± 17,71 bpm, que caracteriza 68% da prevista pela idade. Somente 12% apresentaram acentuada queda de oxigenação no TC6M e 40% apresentaram frequência cardíaca de recuperação no primeiro minuto inferior a 15 bpm. Conclusão: Os pacientes com OAJ, que foram avaliados pelo TC6M, apresentaram baixa capacidade funcional e descondicionamento físico.* ***Nível de Evidência III, Estudo de Caso Controle.***

## INTRODUCTION

 Knee osteoarthritis (KOA) affects about 250 million people worldwide. The condition is inflammatory and can progressively affect the joint, causing joint degeneration. The disease mainly affects women, individuals with obesity and people over 50 years old. ^1^^,^^2^ In the progression of KOA, there may be complaints of disability when performing activities of daily living (ADL) with a progression to physical dependence. ^1^^,^^3^

 KOA has known osteoarticular causative factors and clinical management is complex and may require surgical treatment. Rehabilitation aims to maintain or improve motor function for the full return to work functions and ADLs, but the orthopedic process may not be achieved due to other limitations and associated comorbidity. ^1^^,^^4^ Thus, knowing the functional capacity from the six-minute walk test (6MWT) may allow one to recognize the cardiovascular condition of patients with KOA and assist in the process goals for full treatment, aiming at the social impact of rehabilitation. 

 The 6MWT is a submaximal test of easy application and reproducibility, and for this reason it has been applied frequently. The test is not only used for cardiorespiratory assessment, as it can stratify cardiovascular and surgical risk in patients with various chronic diseases. ^5 ,6^ Thus, the objective of this study was to know the functional capacity of patients affected by KOA from the completion of the 6MWT. 

## MATERIALS AND METHODS

This cross-sectional study was conducted in a tertiary public hospital in the city of São Paulo, after being approved by CAAE under protocol 39890620.5.0000.5479. Data collection took place from January to December 2021.

Patients with a diagnosis of KOA, aged ≥ 18 years, both sexes, randomly referred to physical therapy were included. Patients with heart diseases and/or decompensated lung diseases, previous neurological diseases and patients who could not walk independently were excluded.

 Patients diagnosed with KOA were initially asked about the eligibility criteria and acceptance to participate, with the presentation of the study and the informed consent form. All participants were informed that regardless of the study they would receive care and would have the anonymity of the information collected ensured. To ensure data reliability and reduce the risk of data breaches, assessments were archived in the *Research Electronic Data Capture* (REDCap) data management system. 

 In the evaluation, gender, age (years), weight (kg), height (cm) and comorbidities were collected. The quality of life questionnaire – WOMAC ( *Western Ontario and McMaster Universities* ) was applied, ^7^ which evaluates the symptoms and level of physical disability in the last 72 hours (score of zero and 96). The WOMAC interpretation points out that the higher the value achieved, the worse the self-perception. The scale that allows self-assessment of pain in the last 24 hours (VAS of 0-100) was also applied. ^8^

 Knee lesions were evaluated from the radiographic image and classified according to the degree of impairment, ^9^ by a blind and previously trained evaluator. 

 Patients were kept at rest for 30 minutes for subsequent measurement of the variables: heart rate (HR, bpm), blood pressure (BP, mmHg), peripheral oxygen saturation (SpO2, %), self-perceived exertion (adapted Borg – 0-10) and pain in the lower limbs (VAS). Subsequently, the 6MWT was performed, ^5^^,^^6^ in which all patients were instructed to walk continuously for six minutes, at the highest possible speed, without running, in a 30-meter corridor. Before and immediately after the test, HR, BP, SpO2, Borg and VAS were collected. After the test was completed, the first minute of post-test rest (7th minute) was followed to verify patient safety and to calculate the double product (multiplication between HR and systolic pressure) and effort variation (Borg). 

 The distance covered in the 6MWT was measured and the predicted distance covered was determined by the formula: Distance predicted in the 6MWT: 622.461 − (years of age) + (61.503 × gender men = 1; women = 0). ^5^^,^^6^

 In addition to measuring the distance of the 6MWT, observing the HR behavior throughout the test allows us to assess whether the participants reach 60% of the maximum HR that is calculated from the age of each participant [maximum HR = 220-age(years)]. ^6^

The statistical analysis of this sample was non-probabilistic by convenience through the database built in the REDCap and transposed to the SPSS program, version 25 for descriptive analysis (absolute and relative), mean and standard deviation.

 In the comparison of the means of the distance covered and calculated, the normality analysis was performed by *Shapiro-Wilk* , and compared with the tests: *t* *Student* and *Wilcoxon* (non-parametric variables) with a significance of 5% (p < 0.05). In the correlation analysis, Pearson was used between the distance walked and the variables: pain (VAS), physical disability (WOMAC), BMI and waist circumference. 

## RESULTS

 Among the 176 patients referred, 12 were excluded for declaring heart disease and/or decompensated lung disease, or for not being able to walk without assistance. A total of 164 patients were evaluated, of which: 2.8% had no joint damage at X-ray; 38.6% had grade 1 lesion; 23.9% grade 2; 22.7% grade 3, and 12.5% grade 4. Regarding BMI, 37% had grade 1 obesity, 24% overweight and 20% grade 2 obesity. Other data on clinical and anthropometric characteristics are available in Table 1 . 

**Table 1.** Clinical and anthropometric characteristics of the sample of 164 patients with knee osteoarthritis.

**VARIABLES**	**MEAN ± SD**
Age (years)	61.89 ± 10.62
Body mass index (kg/m ^2^ )	32.33 ± 5.67
Abdominal circumference (cm)	101.29 ± 12.11
Female	81.10% (133)
Comorbidities	67.07% (110)
Cardiac disorders	12.80% (21)
Diabetes Mellitus	21.34% (35)
Dyslipidemia	27.43 (45)
Systemic arterial hypertension	56.09 (92)
Obesity	65.85% (108)
Pain at 24 hours (VAS 0-100)	47.74 ± 29.27
Quality of life (WOMAC 0-96)	58.70 ± 19.34

SD: standard deviation; VAS: visual analog scale; WOMAC: Western Ontario and McMaster Universities (questionnaire).

WOMAC, after categorization, presented 6.1% of the sample with little limitation; 25.6% with moderate; 50.6% intense limitation and 17.7% a lot of limitation due to the repercussions of KOA.

In the 6MWT evaluation (n = 164), 10 patients interrupted the test referring pain in the lower limbs. The HRmax reached at the 6th minute was lower than the calculated HRmax (which was expected for the population) with p < 0.001.

**Figure 1.**
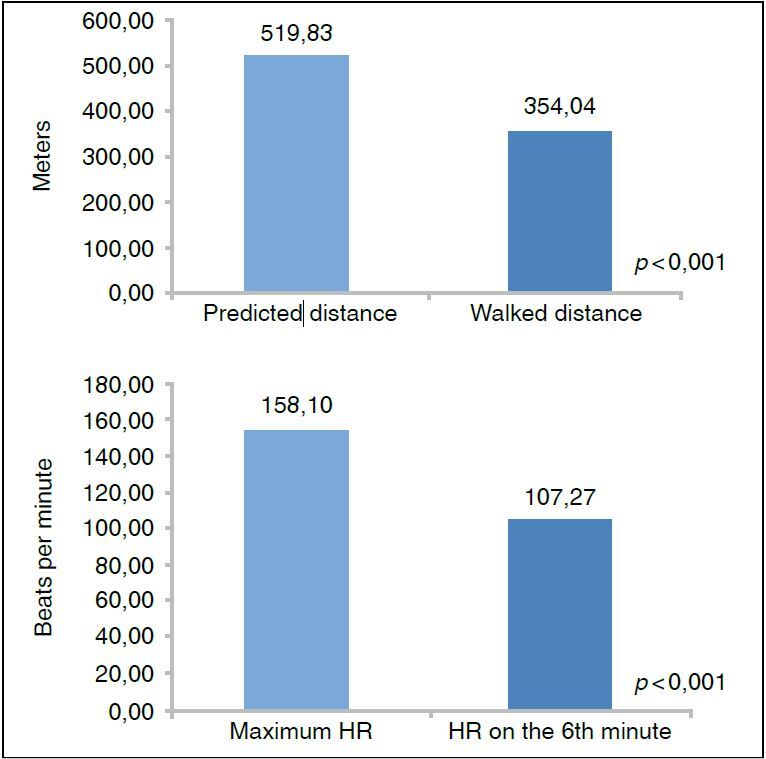
Comparison between averages of the distance covered and predicted, and heart rate achieved and calculated (n = 164).

 The distance covered was shorter than predicted (p < 0.001) according to reference values for the Brazilian population ^10^ and are shown in Table 2 . In this sample, patients reached 67.90% of the predicted distance, which represented a reduction of 165.78 meters (on average) between the predicted value and the path traveled ( Figure 1 ). 

The HR achieved presented a mean of 68.07% of the maximum HR with 9.14% of the sample exceeding 85% of the maximum HR, and 25.60% not reaching 60% of the maximum HR. The difference between HR at the 6th and 7th minute (autonomic nervous system response to physical effort) was less than 15 bpm in 39.63% of patients at the 7th minute.

SpO2 had a variation between rest and the 6th minute greater than 4% in 12.19% of patients during the test. The double product presented an increase of 64.6% in physical demand, and only 4.26% doubled the effort evaluated by this variable. The self-reported effort measured by the Borg was higher after the 6MWT (p < 0.001), as well as the VAS in the lower limbs (p < 0.001) and the double product (p < 0.001).

The correlation of the distance walked with WOMAC was negative (r −0.409, p < 0.001), as well as in the 24-hour VAS (r −0.190, p = 0.015), BMI (r −0.163, p = 0.037), and waist circumference (r −0.079, p = 0.316).

**Table 2.** Evaluation of the functional capacity of patients with knee osteoarthritis by the six-minute walk test.

**VARIABLES**	**MEAN ± SD**
Expected distance (m)	519.82 ± 32.03
Distance travelled (m)	354.03 ± 102.03
HR rest	80.17 ± 11.53
HR 6th minute	107.27 ± 17.71
Maximum HR	158.82 ± 10.62
HR recovery	88.15 ± 13.91
HR difference between 6th and 7th minute	19.12 ± 14.70
SpO2 rest	95.71 ± 2.06
SpO2 6th minute	93.82 ± 3.23
SpO2 difference between rest and 6th minute	−1.88 ± 2.53
Resting systolic BP	129.84 ± 12.70
Systolic BP 6th minute	149.81 ± 19.89
Double Product rest	10,422.77 ± 1,901.27
Double Product 6th minute	16,143.29 ± 3,813.91
Respiratory effort (Borg) rest	0.68 ± 1.54
Respiratory effort (Borg) 6th minute	4.34 ± 2.46
Lower limb effort (Borg) rest	1.73 ± 2.62
Lower limb effort (Borg) 6th minute	5.59 ± 2.50

SD: standard deviation; HR: heart rate; SpO2: peripheral oxygen saturation; BP: blood pressure.

## DISCUSSION

 The treatment of KOA involves a series of changes in lifestyle habits, including measures such as weight control, physical activity and physical therapy, oral and intrarticular medications, nutritional supplements, and even joint cleaning measures via arthroscopy, osteotomy or knee arthroplasty. ^2^^,^^11^^-^^13^ Thus, the look at these patients has changed over time, with conservative treatment being the first choice today. ^2^^,^^13^ One must know the functional capacity, conditioning and physical limitations to provide much more than rehabilitation focused only on the affected joint, as proposed in this study, which sought to observe functional capacity with a simple and low-cost test such as the 6MWT. 

 Functional assessment in KOA is important, as pain is an inhibitory symptom. ^14^ This inhibition can negatively impact the patients’ ability to perform daily living activities. Over time, the individual tends to become more sedentary, and further impair their quality of life. ^10^^,^^13^ The 6MWT is a test that allows the observation of gait as a condition of independence, considered as one of the most important. ^6^^,^^15^ Therefore, how to assess the quality of life and the patient within their limitations, without evaluating their independence, gait and functional demand? 

 Evaluating functional capacity allows the identification of physical fitness, cardiovascular response to exertion, gait performance evaluation according to age and sex, allows the evaluation of risk and prognosis in various clinical conditions, such as cardiovascular and pulmonary diseases, and can even assess morbidity and mortality. ^5^^,^^6^^,^^15^^,^^16^

 In the search for the best tools for assessing functional capacity in patients with KOA, the 6MWT and the 40-meter walk test are described. ^17^ The 6MWT is a clinical test that verifies the distance walked and measures cardiorespiratory variables, which allows a more complete assessment of cardiovascular and respiratory condition. ^5^^,^^6^ The 6MWT is commonly used to measure functional capacity before and after clinical trials. ^5^^,^^6^ However, meta-analysis shows that studies often assess the deficit of function by questionnaires. ^17^ This change in evaluation, with the implementation of the 6MWT, allows us to verify, in addition to the distance walked, physical conditioning and therapeutic effect. ^5^^,^^6^

 The assessment should also contain characteristics such as age, sex, BMI, waist circumference, economic and educational status, and presence of comorbidity, as already described by other authors. ^10^^,^^18^

 What can be seen, in this unicentric sample, was the prevalence of a population aged between the fifth and seventh decade of life, females with high BMI and increased waist circumference, presenting moderate to intense pain by VAS and intense or very intense physical disability by WOMAC. ^10^^,^^18^^,^^19^ The sample characteristic is similar to other studies. However, only a low negative correlation was found between the distance covered and WOMAC. 

 This self-perception of disability may be the result of the decrease in natural muscle strength of age, functional decline resulting from the reduction of ADLs throughout the evolution of the disease and/or the consequence of comorbidity related to OAJ. ^4^^,^^12^^,^^17^^-^^19^ In general, these results on functional capacity are only a picture of what is found when evaluating patients with KOA. In the 6MWT, patients on average did not reach 70% of the predicted distance. Our data indicate that pain (VAS) and physical disability (WOMAC) were less evident than the physical deconditioning observed in this group with obesity and cardiovascular comorbidities. 

 When evaluating patients, it cannot be concluded that KOA is a consequence of obesity, sex and age, or vice versa, this is a cross-sectional study. But it is known that functional disability is not always linked to articular lesions visible on radiographic imaging. ^1^^,^^8^^,^^14^

The functional analysis found in this sample leads us to the second question: can the performance deficit in the distance traveled be explained only by the presence of KOA?

 It was found that a quarter of the sample did not reach 60% of the maximum calculated HR, that is, 25% of the patients did not reach a submaximal effort. And the average heart rate endorses functional limitation and cardiorespiratory deconditioning. The SpO2 evaluation throughout the test showed a reduction greater than 4% from the initial value in some patients, conferring a greater risk to them. ^5^^,^^6^ Finally, in the minute of recovery, when evaluating how many beats reduced in this time interval, more than one third of the sample did not show a reduction of even 15 bpm, which in diseases such as pulmonary hypertension, heart failure and obstructive pulmonary disease demonstrate a high risk of death and alteration of the autonomic system in the control of HR. ^16^

 The SD variation shows that there was an increase in physical effort with a mean of 50% above baseline. The distance covered was low compared to the calculated distance. And finally, the maximum HR reached close to 60% of the calculated maximum. Thus, what can be verified is that there is an important physical deconditioning, in addition to the reduced functional capacity in this sample, with most patients presenting radiographic classification of grade I joint injury. Therefore, little pain can be attributed to this deconditioning. Obesity and waist circumference have also not been shown to be important in the analysis of correlations, as seen by other authors. ^18^^,^^19^ The presence of cardiovascular diseases may demonstrate that the general condition of the sample is more relevant than joint disease, and in the treatment of KOA, attention should be paid to the indication of exercise-based physical therapy, weight reduction and control of comorbidities. ^13^^,^^18^^-^^20^ Surgical procedures may be necessary throughout the lives of these patients, ^11^ and there may be risks related to the cardiorespiratory condition and comorbidities to perform these procedures. ^15^^,^^16^ Understanding the cardiorespiratory condition and limitations in functional capacity may allow the performance of treatment in a safer way, and to treat the patient in a functional way. ^5^^,^^6^^,^^13^^,^^15^^,^^16^ That is, this evaluation can help in the performance of therapeutic exercises and minimize risk in surgical treatment, ^15^^,^^16^ since we know that advanced age, high BMI and the presence of cardiovascular comorbidities may represent a potential risk to patients who require surgical procedures. 

## CONCLUSIONS

Patients with KOA who were evaluated by the 6MWT have low functional capacity and physical deconditioning.
